# Parliament and the Profession

**Published:** 1917-06-02

**Authors:** 


### PARLIAMENT AND THE PROFESSION.

### Proposed Military Venereal Hospital.

Mr. Macpherson informed Mr. Morton that he was aware that the local authorities were against the use of the military hospital huts at Cromarty for venereal cases, and that the whole question is now being considered.

### Medical Inspection of School Children.

The President of the Board of Education, Mr. Fisher, explained to Sir Francis Blake that the war had necessarily stopped a rapid development of school medical service. The need for co-operation between the service and agencies for child welfare and similar organisations had been frequently emphasised in the reports of the Board's Chief Medical Officer, and Mr. Fisher had no reason to anticipate that the development of the work would not be resumed as soon as normal conditions were restored.

### Epileptic Soldiers.

Mr. Macpherson informed Mr. Hogge that an Army Order, issued on December 9 last, provided that a soldier suffering from epilepsy will not be discharged on that account alone, unless his incapacity is such as to unfit him for any military employment suitable to C 3 men. He will be reclassified in a category from which men are eligible for transfer to Class P or P (T.) of the Reserve.

### Promotion of Medical Officers (Territorial Force)

In answer to a question by Mr. Lynch, Mr. Macpherson stated that it is not a fact that all promotions in the Royal Army Medical Corps (Territorial Force) are refused on the grounds that promotions in this branch of the Service are in abeyance. When suitable officers with the requisite qualifications, service, and experience are available promotions are made. Only when such officers are not available for command are regular or temporary officers of the R.A.M.C. brought in. Promotion in the R.A.M.C. (Territorial Force) is now under consideration by a special committee.

### R.A.M.C. (Professional Fees).

Mr. Forster informed Sir G. Toulmin that he was not aware of any reason why any fee ordinarily payable by a life association should not be charged by an officer serving in a military hospital for making a report in respect of a patient who had died whilst under his charge.

### Entertainments to Wounded Soldiers.

Mr. Nield asked the Under-Secretary for War whether he was aware that wounded soldiers in hospital in London have accepted invitations to entertainments provided by registered alien enemies. Mr. Macpherson said that no instances had come under his notice, but that he should regard the practice as objectionable.

### Naval Officers with Tuberculosis.

Dr. Macnamara said that the facts as stated by Mr. Mooney in a question, respecting naval officers with tuberculosis, were generally correct. Out of the full pay allowed to an officer who is sent to a hospital or sanatorium he has to meet doctors' bills and expenses of treatment. Arrangements are, however, provided for the reception of all such cases, after invaliding, into sanatoria at the public expense. It is recognised that the treatment so given is not what should be supplied to our naval officers, and steps are being taken to remedy this defect.

